# Crosstalk between ferroptosis and miRNA in type 2 diabetes mellitus and possible therapeutic targeting

**DOI:** 10.1186/s40001-025-03178-y

**Published:** 2025-10-16

**Authors:** Hebatallah M. Saad, Esraa A. Salem, Omnya Elhussieny, Tasnim S. Waheeb, Abeer E. Elsayed

**Affiliations:** 1 Department of Pathology, Faculty of Veterinary Medicine, Matrouh University, Marsa Matruh, 51744 Egypt; 2 Department of Medical Physiology, Faculty of Medicine, Menoufia University, Shebeen ElKom, 32511 Egypt; 3 Department of Histology and Cytology, Faculty of Veterinary Medicine, Matrouh University, Marsa Matruh, 51744 Egypt; 4 Department of Biochemistry, Faculty of Science, Alexandria University, Alexandria, 21511 Egypt; 5 Department of Physiology, Faculty of Veterinary Medicine, Matrouh University, Marsa Matruh, 51744 Egypt

**Keywords:** Ferroptosis, T2D, MiRNA, Glucose, GPX4

## Abstract

Type 2 diabetes (T2D) accounts for over 90% of diabetes mellitus and is characterized by peripheral tissue insulin resistance, a defective compensatory insulin secretion, and reduced insulin output from pancreatic *β*-cells. T2D is a complex metabolic syndrome involving multiple cell types within multiple organs, such as the liver, muscle, adipose tissue, and pancreas. Because the adult human endocrine pancreas does not have regenerative capability, understanding of the pathogenesis of T2D is vital for working out successful strategies for the delay or arrest of disease development. Newly, ferroptosis, an iron-dependent, regulated cell death, has emerged as a significant promoter of the pathogenesis and development of T2D. Ferroptosis is distinguishable from apoptosis, autophagy, and necroptosis, and is characterized by the accumulation of iron, lipid peroxidation, and suppression of glutathione peroxidase 4 (GPX4). Ferroptosis in pancreatic β-cells results in the defective secretion of insulin. The labile iron pool (LIP), particularly Fe^2^⁺, enhances the formation of reactive oxygen species (ROS) during the Fenton reaction, thereby leading to ferroptosis. Recent empirical studies have revealed an exquisite regulatory interaction between ferroptosis and microRNAs (miRNAs), with the implication being that miRNAs play a central role in the regulation of ferroptosis during T2D. Two-way regulation of ferroptosis by miRNAs has been highlighted herein, with special focus on new insights and the speculation on the potential of using inhibition of ferroptosis as a strategy for treatment. Therapeutic approaches targeting ferroptosis include the use of ferroptosis inhibitors, such as Ferrostatin-1 and Deferoxamine, and miRNA-guided therapy that regulates iron homeostasis and lipid peroxidation. Such interventions may find practical applications in sustaining *β*-cell function and stimulating insulin secretion in diabetic patients. In conclusion, understanding the molecular mechanisms that regulate ferroptosis and identifying specific drugs targeting ferroptosis and associated miRNAs may unlock novel and effective therapies for individuals with T2D.

## Introduction

One of the earliest illnesses to be documented was diabetes, which dates back to an Egyptian book written around 1500 before Common Era [1]. The incidence of Type 2 diabetes (T2D) has significantly increased since 1960, primarily due to sedentary lifestyles and excessive calorie intake, particularly from diets high in saturated fat and carbohydrates. This has led to obesity and T2D, especially in young and old people and ethnic communities with higher genetic risk. About 38.4 million Americans, or 11.6% of the country’s total population, have diabetes as of 2021; 29.7 million of these cases are diagnosed, while 8.7 million are not [2]. Recent estimates indicate that by 2050, the number of Americans with diabetes will have more than doubled, potentially affecting up to 33% of the adult population [3].

Ferroptosis is a unique and newly discovered kind of cell death that has been connected to several illnesses, such as lung cancer [4], infections [5], inflammatory bowel disease [6], diabetes [7], neurological disorders [8], and acute kidney disease [8]. It is characterized by iron buildup, lipid peroxidation, excessive reactive oxygen species (ROS), and depletion of glutathione. Ferroptosis is considered a non-apoptotic controlled cell death process as it does not require caspases (aspartate-specific cysteine proteases). The process is triggered by an iron-dependent buildup of ROS, followed by the peroxidation of membrane polyunsaturated fatty acid phospholipids, causing significant damage [9]. Ferroptosis differs from other programmed cell death due to smaller mitochondria, rupture of the outer mitochondrial membrane, preservation of the plasma membrane, normal nuclear size, and lack of chromatin condensation [9–11].

Ferroptosis significantly contributes to the development and progression of T2D. Iron metabolism plays a role in various aspects of glucose metabolism, including insulin production [12], hepatic metabolism [12], and lipid metabolism [13] while sustaining blood glucose homeostasis across multiple organs and tissues. Therefore, understanding the relationship between ferroptosis and diabetes may result in the development of novel pharmaceutical therapies. Thus, the purpose of this review is to offer fresh insights into possible therapeutic targets and diagnostic biomarkers that could enhance diabetes care and lessen its consequences. In addition, the growing significance of epigenetic regulators, including microRNAs and N6-methyladenosine (m6A), in regulating ferroptosis-related gene expression in type 2 diabetes will be emphasized.

## Type 2 diabetes mellitus (T2D)

### Overview of type 2 diabetes mellitus (T2D)

T2D, earlier referred to as diabetes of adulthood, is distinguished by elevated blood glucose levels, insulin resistance, and inadequate production of insulin [14]. Common symptoms include exhaustion, more frequent urination and thirst, unexplained weight loss, increased hunger, a burning sensation in the body, and sores that do not heal [15]. Furthermore, long-term consequences and complications from elevated blood glucose levels include coronary artery disease, stroke, and diabetic retinal degeneration, which can cause blindness, kidney damage, and inadequate circulation in the lower extremities which may result in amputations [16]. Moreover, the sudden development of hyperosmolarity and hyperglycemic status [17, 18].

T2D is primarily caused by obesity, a sedentary lifestyle, and hereditary variables, which are accountable for the altered glucose homeostasis observed in T2D [19]. In addition, T2D has been linked to gut dysbiosis, such as *Bacteroides vulgatus* and *Prevotella copri* [20, 21]. Approximately 90% of occurrences of diabetes are T2D, and the remaining 10% are mostly caused by type 1 diabetes and pregnancy-related diabetes [22]. Diabetes is diagnosed using blood tests, such as glycated hemoglobin (HbA1C) levels, glucose tolerance testing, and plasma glucose levels during fasting [23]. T2D can be prevented by maintaining a healthy weight, getting regular exercise, and eating a balanced diet rich in fruits and vegetables and low in harmful fats and carbs [24], and if blood sugar levels are not sufficiently reduced, the medicine metformin is usually advised [25].

Alarming findings from epidemiological statistics point to a concerning future for T2D. The International Diabetes Federation (IDF) reports that among diabetic patients, diabetes killed 4.2 million people in 2019, and that 463 million people aged and increased to 700 million diabetic patients in 2045 [26]. The development of T2D is driven by many risk factors, including genetic predisposition, involving dysfunction in liver function and insulin secretion. The need for better risk factors is highlighted by the excessively high glucose levels caused by this malfunction, which is further worsened by β-cell failure [27, 28]. Improving modifiable variables such as obesity, inactivity, and bad diets can prevent many instances, whereas non-modifiable ones such as ethnicity and family history contribute to the prevalence [29, 30].

### Pathophysiology of type 2 diabetes (T2D)

T2D is a long-term metabolic condition characterized by tissue insulin resistance (IR), insufficient insulin compensatory secretion response, and insufficient insulin secretion by islet *β*-cells of the pancreas [31, 32]. The liver, kidneys, brain, skeletal muscle, adipose tissue, small intestine, and pancreas (*β*-cells and *α*-cells) are among the organs implicated in the development of T2D [33]. Adipokine dysregulation, inflammation, and alterations in gut microbiota have been identified as significant pathophysiological contributors, according to evolving research [34].

**Fig. 1:**
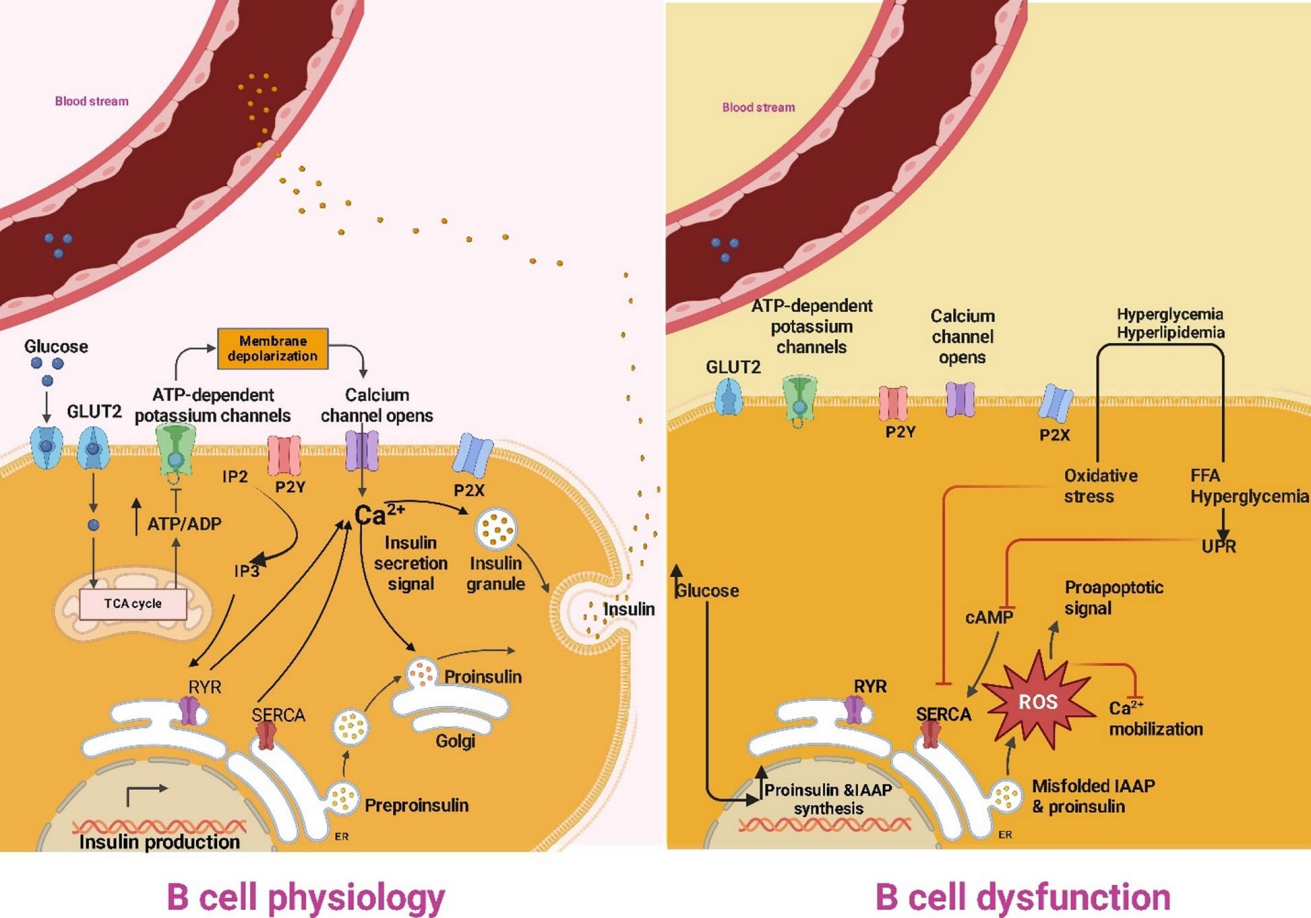
Mechanisms of *β*-cell insulin secretion under normal and diabetic conditions. The insulin secretion from the *β*-cells, under normal settings (**A**), is triggered by high glucose concentration. Glucose enters the cell through glucose transporter 2 (GLUT2). The adenosine triphosphate (ATP)/adenosine diphosphate (ADP) ratio rises as a result of glucose catabolism. ATP-dependent potassium channels are shut, causing membrane depolarization with subsequent stimulation of phospholipase C (PLC) activity and activation of voltage-dependent Ca^2+^ channels. The latter permits the entry of Ca^2+^, which in turn causes the exocytosis of insulin. Ca^2+^ mobilization and insulin secretion are facilitated by additional Ca^2+^ channels, such as purinergic receptor X and Y (P2X and P2Y), Sarco-endoplasmic reticulum Ca^2+^-ATPase (SERCA), and ryanodine receptor channel (RYR). Furthermore, PLC hydrolyzes PIP₂ (phosphatidylinositol 4,5-bisphosphate) into DAG (diacylglycerol) and IP₃ (inositol 1,4,5-trisphosphate). IP₃ binds to IP₃ receptors (IP₃R) on the endoplasmic reticulum (ER), causing Ca^2^⁺ release from ER stores into the cytoplasm. In contrast, hyperglycemia and lipotoxicity (**B**) induce oxidative stress and ER stress, triggering unfolded protein response (UPR), reduced Ca^2^⁺ signaling, accumulation of misfolded proteins, and activation of proapoptotic pathways. These disturbances stimulate proapoptotic signaling, resulting in β-cell dysfunction and impaired insulin secretion. ROS: reactive oxygen species; FFAs: free fatty acid

In pancreatic *β*-cells, insulin biosynthesis begins in the endoplasmic reticulum, where pre-proinsulin undergoes conformational changes to form proinsulin [35], is then cleaved into insulin and C-peptide in the Golgi apparatus. The produced insulin is stored in secretory granules [36]. The main cause of insulin release is elevated blood glucose levels. It is important to remember that hormones, fatty acids, and amino acids are other substances that might cause the release of insulin [37]. A solute carrier protein called glucose transporter 2 (GLUT2), a solute carrier protein, allows *β*-cells to absorb glucose. Once glucose catabolism the ATP/ADP ratio is increased, which also closes potassium channels in the plasma membrane. Ca^2+^ enters the cell through membrane depolarization and the opening of voltage-dependent Ca^2+^ channels. As a result, secretory insulin-containing granules are primed and fused, raising the intracellular Ca^2+^ concentration and causing insulin exocytosis (Fig. 1A) [37, 38].

Genetic susceptibility and overnutrition states, i.e., chronic hyperglycemia and hyperlipidemia, are the causes of insulin resistance and low-grade chronic inflammation. These metabolic stressors induce endoplasmic reticulum (ER) stress via activation of the apoptotic unfolded protein response (UPR), posing toxic pressure on pancreatic β-cells. Furthermore, β-cells increase proinsulin and islet amyloid polypeptide (IAPP) synthesis after chronic hyperglycemia to enhance the intracellular retention of misfolded insulin and IAPP in the ER. Oxidative protein folding catalytically increases this misfolding, generating ROS that further destabilize cellular structure. The resulting stressful environment attracts macrophages that trigger local inflammation in the islet. To satisfy the increased demand for metabolism, β-cells attempt to secrete extra insulin. This adaptive response, though, can destroy islet architecture, impair cell-to-cell communication, and disrupt concerted release of insulin and glucagon and thereby worsen hyperglycemia during pathologic states. Ultimately, insulin secretory malfunction due to impaired insulin biosynthesis, structural flaws in insulin, and impaired exocytosis represents an important mechanism of β-cell failure and T2D (Fig. 1B) [39]**.**

Even when hyperglycemia is managed, diabetic problems may still arise, because hyperglycemia causes mitochondria to produce too many ROS [40]. When proper glycemic control is started very early, the damage caused by oxidative stress due to hyperglycemia can be avoided; nevertheless, if inadequate control continues for an extended period, it is difficult to reverse [41, 42]. Hyperglycemia, oxidative stress elevation, and excessive advanced glycation end products (AGEs) production are all related to the initial phases of T2D. The respiratory chain’s components experience chronic protein glycation as the illness worsens, which, when combined with the damaged DNA of mitochondria, can cause a series of events that are independent of hyperglycemia and result in a synergy between oxidative stress and AGEs [43]. Through receptors, the consequences of this metabolic disequilibrium trigger inflammatory processes, low-grade inflammation, and nuclear factor kappa B (NF-κB) [44]. Insulin resistance is a feature of T2D, but the primary mechanisms by which it is associated with ferroptosis are increased oxidative stress and lipid peroxidation. Hyperglycemia and impaired insulin signaling contribute to the accumulation of ROS, which increases the susceptibility of cells to ferroptosis via iron-dependent lipid damage mechanisms [45].

## Ferroptosis

### Overview of ferroptosis

Ferroptosis is a recently identified kind of iron-dependent cellular death marked by iron accumulation and an accumulation of lipid peroxidation. In 2003, Dolma, Lessnick [46] first found that the chemical erastin was found, which demonstrated specific lethality towards cancer cells expressing renin–angiotensin system (RAS); nonetheless, it was observed that the cells succumbed by a mechanism distinct from conventional programmed cell death. As this research progresses, Dixon, Lemberg [47] first developed ferroptosis in 2012, an iron-dependent variant of regulated cell death (RCD) characterized by unique morphological and biochemical attributes relative to other forms of cell death. Ferroptosis is a specific type of cell death triggered by the presence of ROS that rely on iron [46], it is a distinct form of controlled cell death that lacks the involvement of caspases, which are crucial in inducing apoptosis by cleaving specific intracellular substrates [48, 49]. Unlike other forms of controlled cell death, such as parthanatos and necroptosis, ferroptosis operates independently of apoptotic effectors, such as caspases, Bcl-2 homologous antagonist/killer (BAK), and Bcl-2-associated X protein (BAX) [50]. Notably, ferroptosis relies on intracellular iron and lipid peroxide buildup, setting it apart from other forms of controlled cell death. Unlike necroptosis, ferroptosis does not require vital components, such as receptor-interacting protein kinase 1 (RIPK1), RIPK3, and mixed lineage kinase domain-like protein (MLKL). Ferroptosis also results in the inhibition of oxidoreductase, particularly glutathione peroxidase 4 (GPX4), which is a scavenger of lipid peroxides [7]. Furthermore, downstream of p53, ferroptosis may function as an intrinsic tumor-inhibitory mechanism in the cancer setting [51]. It is still debatable whether or not small molecules of ferroptosis stimulants can be employed to specifically kill cancer cells with anomalies in the RAS–RAF–MEK pathway [46, 52]. Furthermore, ferroptosis is linked to damaged mitochondria in specific cells, such as kidney cells, which exhibit smaller organelles, no longer visible mitochondrial cristae, and ruptured outer membranes [53].

Consequently, the regulation of ferroptosis is intricately linked to the metabolism of iron, lipids, amino acids, and glutathione. In the last 10 years, a growing body of research has substantiated the notion that ferroptosis is significantly involved in the pathophysiology of T2DM and associated consequences [54–56].

### Mechanism of ferroptosis

#### Iron metabolism and ferroptosis

**Fig. 2:**
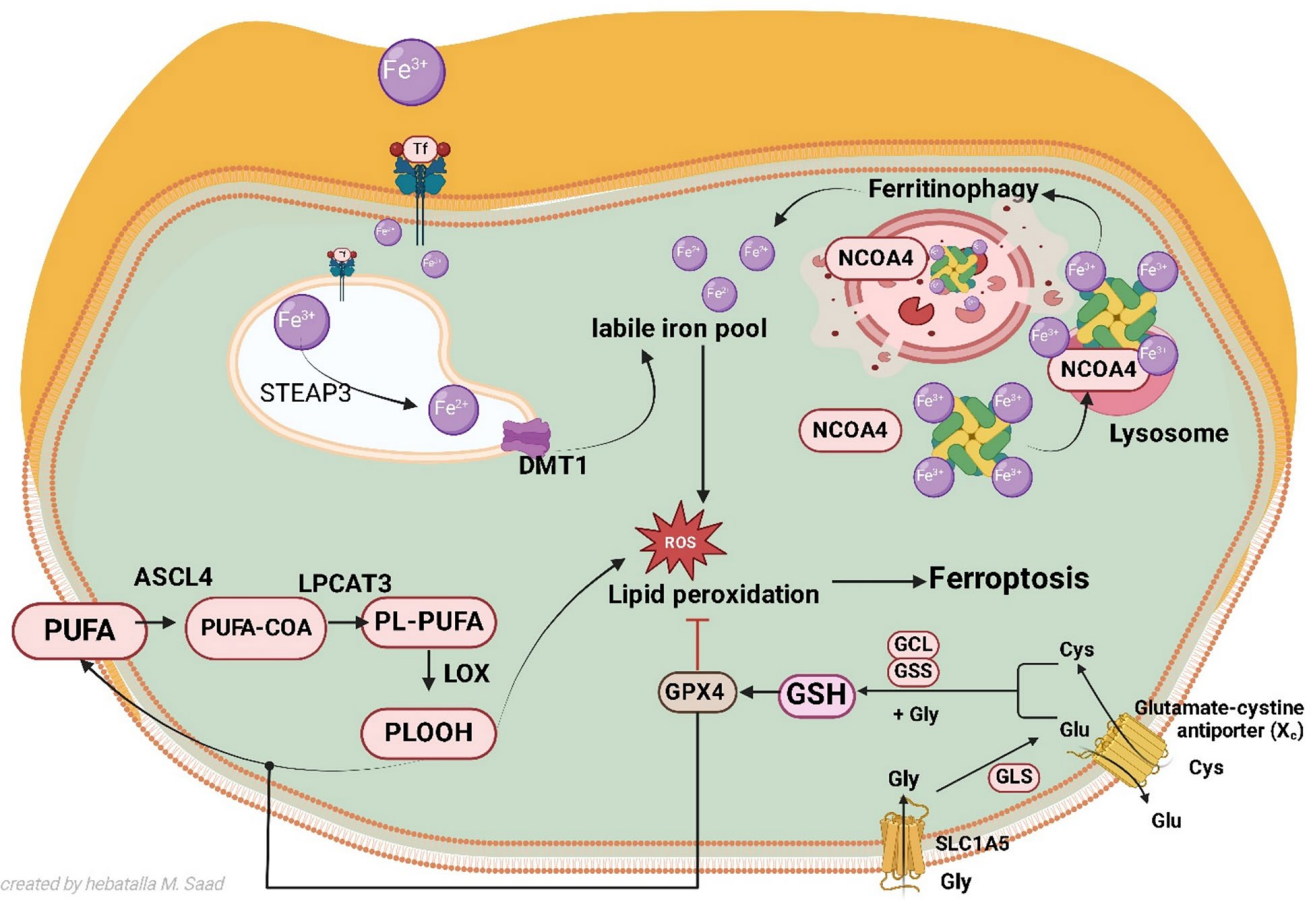
Iron metabolism, ferroptosis pathway, and regulatory miRNAs. Iron overload and ferritinophagy expand the labile iron pool, ROS generation triggers lipid peroxidation, and impaired GPX4/GSH antioxidant defense accelerates *β*-cell death. Mechanisms of ferroptosis involving iron, lipid, and amino acid metabolism. Excess Fe^2^⁺ accumulation, lipid peroxidation, and reduced GPX4/GSH activity promote ferroptosis, while ferritinophagy contributes to labile iron pool expansion. ACSL4: acyl-CoA synthetase long-chain family 4, DMT1: divalent metal transporter 1; GPX4: glutathione peroxidase 4, GSH: glutathione, LOX: lipoxygenase, LPCAT3: lysophosphatidylcholine acyltransferase 3, NCOA4: nuclear receptor coactivator 4, PUFA: polyunsaturated fatty acid, SLC7A11: cystine/glutamate antiporter, PE: phosphatidylethanolamine, STEAP3: six-transmembrane epithelial antigen of prostate 3 metallo-reductase, SLC7A11: solute carrier family 7 member 11, TF: transferrin, TFR1: transferrin receptor

Iron is a crucial trace element in our bodies. Iron metabolism encompasses four primary aspects: absorption, storage, utilization, and excretion [57]. Iron is transported in the blood bound to transferrin (TF) after absorption from food, primarily in the Fe^3^⁺ form. Cells can absorb circulating iron through the TF/TFR-1 transport system (transferrin receptors) via endocytosis. Divalent metal transporter 1 allows Fe^3^⁺ to enter the cytoplasm after conversion to Fe^2^⁺ by six transmembrane epithelial antigens of prostate 3 (STEAP3) inside the endosome. The reduced Fe^2^⁺ is transported to the cytoplasm by divalent metal transporter 1 (DMT1) and participates in various physiological and biochemical activities, such as DNA biosynthesis, oxygen transport, and metabolic pathway regulation [58]. Upon saturation of the iron-binding complexes, surplus Fe^2+^ accumulates in an unstable iron reservoir. The Fenton reaction, catalyzed by excess Fe^2^⁺, generates hydroxyl radicals, leading to oxidative stress and ultimately ferroptosis through the accumulation and increase of ROS [59] (Fig. 2).

In the extracellular environment, pancreatic *β* cells secrete hepcidin, which binds to transferrin and facilitates its internalization [60, 61]. Research indicates that transferrin facilitates a positive feedback mechanism for iron control during glucose-stimulated insulin production [62]. Fe^2+^ in the Labile Iron Pool (LIP) participates in insulin production. Iron is present in nearly all intracellular organelles; however, it is mostly utilized by mitochondria, the main site of cellular iron metabolism. Production of heme and Fe–S clusters, utilized in electron transport proteins, occurred in the mitochondrial organelle [45].

#### Amino acid metabolism

Glutathione (GSH) is a tripeptide composed of glycine, cysteine, and glutamic acid, featuring Y-amido-hexapeptide and sulfhydryl groups, and serves as a crucial antioxidant [63]. GSH operates intracellularly as an essential substrate for GPX4 [64]. The cell membrane has a type of heterodimer referred to as System Xc-, consisting of Solute Carrier Family 7 Member 11 (SLC7A11) and Solute Carrier Family 3 Member 2 (SLC3A2) [65]. The system Xc- facilitates the transfer of extracellular cystine to intracellular glutamate in a 1:1 ratio, subsequently leading to the synthesis of GSH from intracellular cystine, supported by the functions of glutamate–cysteine ligase (GCL) and glutathione (GSS) [64]. The majority of mammalian cells mediate cysteine absorption by SLC7A11, subsequently undergoing a reduction reaction that consumes nicotinamide adenine dinucleotide phosphate (NADPH) to generate cysteine. Cysteine can be synthesized from methionine via the trans-sulfuration route [66]. In addition, the GPX4 coenzyme of GSH facilitates the conversion of phospholipid peroxides into phospholipids, thus protecting cells from ferroptosis [11, 66]. Thus, inhibition of System Xc⁻ increases susceptibility to ferroptosis by lowering intracellular cysteine and GSH levels [67] (Fig. 2).

Moreover, the modulation of ferroptosis can be affected by mitochondrial tricarboxylic acid (TCA), and the suppression of the TCA cycle in mitochondria can obstruct the voltage-dependent channel 2/3 (VDAC2/3) to protect cells against ferroptosis [68].

#### Lipid metabolism

Polyunsaturated fatty acids (PUFAs) are essential constituents of the phospholipid bilayer and play a vital role in modulating cell membrane fluidity. However, PUFAs (including arachidonic acid and epinephrine) are susceptible to the Fenton reaction, resulting in the generation of excessive peroxides that disrupt the phospholipid bilayer structure, hence undermining cell membrane functionality [69]. PUFAs interact with coenzyme A to generate acyl-CoA via the catalytic activity of acyl-CoA synthetase long-chain family member 4 (ACSL4). Lysophosphatidylcholine acyltransferase 3 (LPCAT3) subsequently catalyzes the conversion of acyl-CoA to membrane phosphatidylcholine (PE) via esterification, yielding PUFA-PE [70]. The oxidation of PUFA-PE by lipoxygenase (LOX) leads to cellular ferroptosis [71] (Fig. 2).

Also, FPT is driven by lipid peroxidation (LPOX) levels, which are only able to happen in situations, where there is either no enzymatic inhibition of phospholipid hydroperoxides (PHL-OOH) or a severely restricted amount of it relative to the rate at which it forms [72]. The process by which LPOX causes ferroptosis completely overlaps with that of iron-dependent lipid peroxidation, which was first described over 50 years ago. Ferrous iron complexes and pre-existing lipid hydroperoxides are catalysts for the oxidative breakdown of polyunsaturated phospholipids, known as membrane LPOX [66, 73]. Thus, reducing PL-OOH to a nontoxic alcoholic substance by GPx4 utilizing GSH as a reducing substrate is solely an enzymatic process capable of stopping the entire peroxidative process [72]. When glutathione-dependent antioxidant systems are deactivated, harmful lipid reactive oxygen species (L-ROS) build up and cause ferroptosis [47, 74]. To put it another way, GPx4’s impact is clear proof that PL-OOH is the source of LPOX. Since PL-OOH is a byproduct of LPOX, it could seem contradictory, but this problem is resolved by keeping in mind that residues of PL-OOH can occur independently of the start and advancement of rapid peroxidative reactions [75]. Without particular obstacles, GPx4 and GSH continue to catalyze the conversion of phospholipid alcohols (PL-OH) into their equivalent PL-OOH, which are created in trace quantities during aerobic metabolism. In addition, lipoxygenases may also create PL-OOH [76], with particular attention paid to lipoxygenase 12/15 (ALOX-12/15), and this appears to be pertinent to FPT [77]. Nevertheless, GPx4 activity regulates this lipoxygenase’s function as well, since it needs lipid hydroperoxides to activate [73]. The LPOX of biological membranes includes the following free radical reactions: initiation, which starts with the removal of a hydrogen atom from polyunsaturated fatty acid by creating free radical, resulting in a phospholipid carbon-centered radical (PL); (ii) chain development, which interact with molecular oxygen to form radical of lipid peroxy PL-OO [78] then reaction between PL-OO and another PL, yielding a phospholipid hydroperoxide (PL-OOH); and (iii) arrest, which is caused by the capture or interactions of radical–radical [79]. Within the framework of this streamlined process scheme, Fe^2+^ from PL-OOH initiates LPO. Fe^2+^ may readily break the weak O–O link in PL-OOH, resulting in the highly reactive alkoxyl radical (PL-O), capable of initiation [78]. Therefore, PL-O initiates LPO, and PL-OO drives propagation [80].

## Role of ferroptosis in T2DM

Ferroptosis is a regulated form of cell death driven by iron-dependent lipid peroxidation. It plays a critical role in pancreatic diseases due to the pancreas’s vulnerability to oxidative stress and iron accumulation [81, 82]. The imbalance of iron and Fe–S clusters FV in *β*-cells leads to mitochondrial iron buildup [83, 84]. Research indicated that pancreatic *β* cells exhibit minimal expression of antioxidant enzymes, including superoxide dismutase (SOD), GSH, and catalase [85]. This can result in ROS generation, stress in the endoplasmic reticulum, ferroptosis, and insulin production failure [86]. In addition, it plays a role in diabetes complications, such as endothelial dysfunction and myocardial ischemia [7, 87].

External stimuli, such as chronic arsenic exposure, induce mitochondrial damage and generate excess mitochondrial ROS, which then trigger mitochondrial ROS-dependent autophagy and ferroptosis, culminating in an elevation of intracellular iron levels. This leads to elevated Fe^2+^ production in pancreatic *β* cells and compromised insulin secretion. Experimental verification showed that the inhibition of the mitochondrial ROS-mediated pathway enhances insulin production from pancreatic *β* cells [88], subsequently causing ferroptosis due to the accumulation of lipid peroxides [89].

In conclusion, ferroptosis, driven by iron overload and lipid peroxidation, contributes to pancreatic β-cell failure due to weak antioxidant defenses. Mitochondrial ROS and iron accumulation impair insulin secretion, linking ferroptosis to diabetes onset and its complications.

## Therapeutic strategies targeting ferroptosis in T2D

Ferroptosis contributes to the development of T2DM and its consequences; therefore, it represents a prospective therapeutic avenue for the treatment and prevention of T2DM and metabolic disorders. This section summarizes various compounds that can suppress ferroptosis and their roles in the metabolic and molecular pathways associated with ferroptosis.

### Pharmacological agents and natural compounds

New insights are provided by studies on ferroptosis and its use in natural resources. However, the therapeutic benefits of herbal remedies are limited by their varied origins and complicated components. To increase medication efficacy and identify the best course of therapy, future research will concentrate on creating natural compounds that act as ferroptosis modulators [90]. The discovery process of ferroptosis and its regulatory processes are examined in this review, which also builds a database of natural products and summarizes their effects on ferroptosis control and their effect on DM (Table 1).

**Table 1:** Drugs and herbal agents that target ferroptosis in type 2 diabetes (T2D)

Category (natural vs pharmaceutical)	Agent	Chemical structure	Mechanism of action	Model of study	Adverse effects	Therapeutic uses	Evidence level	References
Natural	Quercetin	It is one of the flavonoids that is most commonly found in plants. (3,3′,4′,5,7-pentahydroxyflavone)	-It reduces oxidative damage in the tissues of the pancreas by scavenging free radicals -It inhibits lipid peroxidation by controlling glutathione peroxidase 4 (GPX4) and maintaining glutathione (GSH) levels -Quercetin decreases the oxidative stress induced by iron by lowering iron levels in the pancreatic islets -It reduces the symptoms of T2D by protecting pancreatic β cells and enhancing insulin production and β cell activity	Mice models	- No possible side effects, - It is safe, but if it is taken in high dosages, thus may cause allergy and gastrointestinal problems	- restoring normal β cell activity by reducing ferroptosis in type 2 diabetes	preclinical	[106]
Pharmaceutical	Erythropoietin (EPO)	A kind of glycoprotein with 165 amino acids that weighs 30.4 kDa	-EPO promotes the survival and development of neurons by exhibiting neuroprotective and neurotrophic effects -EPO lowers fasting blood sugar levels -EPO helps control ferroptosis-related proteins by lowering iron levels and lipid peroxidation -EPO improves mental performance	-Animal mice model -Cell culture PC12 model	-Safe but require monitoring Especially at high dosages or in specific groups, hypertension, and thromboembolic complications are possible negative consequences. [112]	EPO is mostly used for the treatment of anemia, particularly in cases with chronic renal disease Potentially neuroprotective roles in neurodegenerative diseases EPO may lessen the cognitive decline brought on by diseases like type 2 diabetes	Preclinical	
Pharmaceutical	Liraglutide	It is glucagon-like peptide-1 (GLP-1), an analog of the 30-amino acids acid hormonal peptide	-Liraglutide alleviates T2D-related NAFLD, presumably via inhibiting ferroptosis and activating the AMPK/ACC pathway which prevents ferroptosis and encourages lipid metabolism - Causing an increase in insulin production by mimicking the actions of GLP-1 and inhibiting the release of glucagon - Increasing fullness by delaying stomach emptying	-Mice model, T2D induction by high-fat diet followed by STZ injection	Liraglutide is usually tolerated without problems, although some notable side effects include Feeling queasy and throwing up	Controlling T2D (enhancing glycemic control) Its capacity to mitigate liver damage and prevent ferroptosis in the setting of T2D suggests that it may improve the condition of the liver in NAFLD	Preclinical	[105]
Natural	Poliumoside	It is derived from Callicarpa kwangtungensis Chun	Poliumoside can interfere with ferroptosis by activating the Nrf2/GPX	-murine model -Invitro stem cells of bone mesenchyma(BMSCs)	–	-Treatment of T2D with bone degradation	Preclinical	[111]

*T2D* Type2 diabetes, *STZ* streptozotocin, *NAFLD* non-alcoholic fatty liver disease

#### Metformin

Among the first-line medicines against diabetes, metformin can inhibit oxidative stress and affect ferroptosis. Although in widespread practice, its chronic administration is marred by gut intolerance and lactic acidosis risk in some populations. The natural flavonoid quercetin showed encouraging antidiabetic activity by mechanisms, such as antioxidant, insulin-sensitising, and ferroptosis-related pathway modulating activity. However, there is sparse human clinical data and low availability (< 1 μM plasma levels) [91]. a synthetic biguanide that improves glucose management in diabetics by activating the AMP-activated protein kinase (AMPK) signaling system, inhibiting gluconeogenesis in the liver and increasing insulin-stimulated peripheral glucose absorption [92]. The liver kinase B1 (LKB1)/AMPK signaling pathway is crucial for glucose regulation [93]. A prior investigation demonstrated that LKB1 and its downstream AMPK inhibited ferroptosis by obstructing the phosphorylation of acetyl-CoA carboxylase 1 (ACC1) and fatty acid synthase (FAS) [94]. Metformin decreased the accumulation of iron in the liver’s cells through the AMPK–ferroportin pathway in a preclinical model of non-alcoholic fatty liver disease [95]. Metformin preserves cells by activating the AMPK system, modulating metabolism, and safeguarding them against degradation and pathogenic alterations at the molecular level. Consequently, we suggest that metformin’s efficacy in enhancing T2D is linked to the suppression of ferroptosis and the mitigation of IR. Metformin’s anti-ferroptosis properties reduced the calcification of vascular smooth muscle cells in a rat model of calcification via nuclear factor erythroid 2-related factor 2 (Nrf2) signal activation [96]. Ferroptosis was also inhibited by metformin’s alteration of the gut microbiota, which was marked by a rise in bacteria that produce gamma-aminobutyric acid (GABA) [97].

#### SGLT2 inhibitors

A family of medications known as SGLT2 inhibitors, such as empagliflozin, dapagliflozin, and canagliflozin, can lower blood glucose levels by preventing the reabsorption of glucose in the proximal tubules. Once-daily dosing is possible with SGLT2 inhibitors, because they are orally bioavailable, have long half-lives (10–13 h), and high absorption rates [98]. Moreover, they lower ferroptosis, protect the heart and kidneys. According to preclinical research, in diabetic mice, the medications improve cardiac function in heart failure models, stimulate tubular kidney function, and increase revascularization. Because of their possible advantages, their use has grown [99, 100]. The decline in ferroptosis is brought about by increasing intracellular glutathione levels and inducing sirtuin-1, both of which strengthen glutathione-dependent glutathione peroxidase 4 [101].

#### Liraglutide

The common medications for T2D, glucagon-like peptide-1 receptor agonists (GLP-1 RA) are known to enhance the balance of glucose and encourage weight loss in people who are overweight. It is administered by injection, because it has poor oral absorption and has a short biological half-life. It may be useful to use it loaded in nanochitosan, which increases the bioavailability by ten times when administered orally [102]. According to preclinical research, liraglutide, a drug used to regulate blood sugar, may lessen ferroptosis, because it reduces the hepatic iron buildup seen in db/db mice, which in turn lessens IR [103]. In addition, another research revealed a decrease in iron deposition in the hippocampus, which improved synaptic plasticity and decreased damage to hippocampus neurons, encouraging the restoration of cognitive function in db/db mice [104]. Furthermore, T2D frequently results in non-alcoholic fatty liver disease (NAFLD), which is impacted by oxidative stress, inflammation, insulin resistance, and heredity. It has been discovered that liraglutide improves NAFLD in mice. According to the study, liraglutide reduced liver tissue damage and enhanced glucose metabolism. In addition, it preserved the viability of liver cells in high-glucose circumstances. According to the results, liraglutide may alleviate NAFLD by blocking ferroptosis and triggering the AMPK/ACC2 pathway [105].

#### Quercetin

Quercetin, a prevalent flavonoid, is found in fruits, vegetables, and medicinal plants and has garnered interest due to its potential benefits in treating metabolic disorders, which are significant global public health concerns. Quercetin has been shown in animal studies to increase insulin secretion, improve insulin resistance, reduce blood cholesterol levels, decrease inflammation, decrease hepatic fat accumulation, and control diseases associated with the gut microbiota. Nevertheless, there are not many human clinical investigations; therefore, more investigation is required to increase bioavailability and confirm its efficacy in people [91]. In T2D diabetic mice, quercetin, an inhibitor of ferroptosis, enhanced insulin secretion and reduced oxidative stress [106]. Research indicates that quercetin administration markedly reinstates GSH levels and SOD activity in pancreatic β cells [106]. According to the study, ferrous-chelating deferoxamine, ferroptosis suppressor ferrostatin-1, and quercetin may all be useful T2D management methods for preventing ferroptosis and pancreatic iron accumulation [106]. The findings suggest that quercetin may have a favorable impact on T2D by decreasing ferroptosis in pancreatic β cells, underscoring its potential therapeutic efficacy in T2D.

#### Erythropoietin (EPO)

Moreover, erythropoietin (EPO) can alleviate cognitive deficits in T2D mice and lower fasting blood glucose. In mice with T2D, EPO also enhanced cognitive performance and decreased hippocampus damage [107, 108]. EPO also controlled the production of proteins linked to ferroptosis and decreased lipid peroxidation and iron levels. These results imply that by reducing iron overload and preventing ferroptosis, EPO may mitigate cognitive impairment linked to T2D [108].

#### Poliumoside

The idea of Traditional Chinese Medicine asserts that Poliumoside (Pol), mostly located in Callicarpa kwangtungensis Chun, improves blood circulation, stimulates Qi movement, and alleviates pain [109, 110]. Pol counteracts ferroptosis by mitigating oxidative stress via the Nrf2/GPX4 pathway in diabetic mice with osteoporosis [111].

## MicroRNAs and epigenetic regulators of ferroptosis

### Interplay between microRNAs and ferroptosis in diabetes development and therapy

MicroRNAs (miRNAs) are small noncoding RNAs, approximately 22 nucleotides in length that have attracted significant research interest. They play a critical role in gene regulation by binding to specific sites in messenger RNAs (mRNAs), typically in the 3′-untranslated region (3′-UTR), which leads to either the degradation of mRNAs or the inhibition of their translation. Due to their exceptional stability compared to the more unstable transcriptome, miRNAs have emerged as potential biomarkers [113]. Research has established that miRNAs are associated with the regulation of iron through various specific targets, with key signaling pathways related to cell death predominantly influenced by miRNAs. Thus, ferroptosis is modulated by the expression levels of certain miRNAs, which target key genes involved in the ferroptotic process. This suggests a well-established connection between iron and miRNAs, leading to a new dual regulation framework for iron-dependent non-apoptotic programmed cell death. In this framework, miRNAs affect the signaling pathways related to iron or their principal regulators, while this type of cell death may, in turn, influence the cellular levels of miRNAs to some degree [114].

Molecular mechanisms of ferroptosis provide a basis for understanding its therapeutic targeting in T2D. The miRNAs have been shown to modulate ferroptosis in diabetic complications. These miRNAs influence key ferroptotic regulators, such as GPX4, SLC7A11, and ACSL4, suggesting their potential as therapeutic targets. According to these molecular insights, the next section explores how the miRNAs affect the ferroptosis regulation, with a focus on diabetic retinopathy and nephropathy.

#### MicroRNA and diabetic retinopathy (DR)

T2D is a progressive condition that, if unmanaged, can result in severe consequences, including macrovascular and microvascular disorders [115]. Diabetic retinopathy** (**DR) is a microvascular condition associated with T2D. It has been discovered that Astragaloside-IV, a naturally occurring substance from Astragalus, downregulates miR-138-5p in retinal pigment epithelial (RPE) cells of the DR model, leading to a boost in the expression of Nrf2, Sirtuin 1 (Sirt1), the antioxidant activity of GPX4, glutamate–cysteine ligase modifier subunit (GCLM) and glutamate–cysteine ligase catalytic subunit (GCLC), suggesting that the miR-138-5p/Sirt1/GPX4 axis helps mitigating ferroptosis and promotes cell survival in RPE cells exposed to high glucose conditions [116].

On the other hand, Zhu and Duan [117] explored that downregulating circ-PSEN1 increases cell survival rates and reduces ferroptosis in adult RPE-19 exposed to high glucose conditions. This suggests that targeting circ-PSEN1 could protect retinal cells from the damaging effects of high glucose levels. In addition, in DR, the miR-200b-3p plays an important role in regulating ferroptosis. It is sponged by circ-PSEN1, which means that circ-PSEN1 can bind to miR-200b-3p and prevent it from exerting its effects. This interaction implies that downregulating circ-PSEN1 could increase the availability of miR-200bp, which has protective effects against ferroptosis by targeting and inhibiting cofilin (CFL2), thereby reducing oxidative stress and cell death. The use of a miR-200b-3p inhibitor led to a significant decrease in GSH levels, an increase in MDA levels, and an elevation of ferrous ion contents. Under high glucose conditions, miR-200b-3p promotes the expression of transferrin receptor 1 (TFR1) while negatively regulating the expression of anti-ferroptosis genes, such as GPX4 and SLC7A11 [117]. In cells where miR-200b-3p is overexpressed, this change exacerbates ferroptosis. Therefore, treatments that lower circ-PSEN1 may indirectly alter the miR-200b-3p/CFL2 axis, improving DR cellular outcomes. The findings suggest that circ-PSEN1 knockdown may reduce ferroptosis. These findings suggested that targeting the circ-PSEN1/miR-200b-3p/CFL2 may reduce ferroptosis in DR [117].

Another work by Huang and Peng [118] used bioinformatics techniques to uncover ferroptosis-related genes in DR. The R software DESeq2 was used to analyze RNAseq data from retinas in DR and healthy controls. The protein–protein interaction network analysis found seven hub genes associated with ferroptosis in DR: HMOX1, PTGS2, EGFR, CAV1, TLR4, MAPK8, and CDKN2A. Upon verification with an alternative data set, only Heme Oxygenase 1 (HMOX1) and prostaglandin–endoperoxide synthase 2 (PTGS2) were identified as differentially expressed between the DR and control groups. In addition, they indicated that hsa-miR-873-5p has been identified as a key microRNA that regulates the expression of HMOX1, which is one of the hub genes implicated in the ferroptosis process within DR. While hsa-miR-624-5p and hsa-miR-542-3p are identified as the primary miRNAs that regulate the expression of PTGS2. MiR-542-3p is particularly noted for promoting the rapid degradation of PTGS2 mRNA, which supports the findings from bioinformatics predictions. Furthermore, HMOX1 and PTGS2 are regulated by 13 and 20 transcription factors, respectively. The involvement of these transcription factors suggests a complex regulatory network influencing the expression of these hub genes. The role of hsa-miR-624-5p in regulating PTGS2 is less clearly defined in the available literature. While it is categorized as a key miRNA influencing PTGS2, specific studies detailing whether it upregulates or downregulates PTGS2 have not been thoroughly investigated. Previous research on hsa-miR-624-5p suggests its involvement in cancer, but its precise impact on PTGS2 in the context of DR remains uncertain and requires further study [119]. These results underscore the need for additional research on the miR-873-5p/HMOX1 axis, specifically, its therapeutic potential and function within larger transcriptional networks that impact iron metabolism and oxidative stress.

Moreover, another study investigated the molecular processes involved in DR by administering high levels of glucose to the RPE. The findings indicated that excess glucose negatively impacts cell viability and proliferation, increases the levels of ROS, and promotes ferroptosis in the cells. The research showed that miR-338-3p targeted the 3’UTR of SLC1A5 for inhibition and degradation, while elevated glucose levels reduced SLC1A5 by enhancing miR-338-3p in RPE cells. By targeting miR-338-3p/SLC1A5, the researchers successfully prevented high glucose-induced ferroptosis in RPE cells [120].

In addition, the study by Liu, Zhang [121] showed that lncRNA zinc finger antisense 1 (ZFAS1) is elevated in high glucose situations, and its suppression diminishes ferroptosis in human retinal endothelial cells. ZFAS1 may function by binding to microRNA-7-5p, therefore, modulating the expression of ACSL4, a crucial component in ferroptosis. The findings of this study indicate that targeting the ZFAS1/miR-7-5p/ACSL4 axis may serve as a viable treatment option for DR. For instance, inhibiting ZFAS1 or upregulating miR-7-5p could reduce ferroptosis, providing a new strategy for preserving endothelial cell health in diabetes (Table 2).

**Table 2:** Classification of ferroptosis-related miRNAs in T2D

miRNA	Function	Target(s)	Ref	Ferroptotic effect
miR-138-5p	Pro-ferroptotic	Sirt1/GPX4 axis	[116]	↑ Ferroptosis
miR-200b-3p	Pro-ferroptotic	GPX4, SLC7A11	[117]	↑ Ferroptosis
miR-338-3p	Pro-ferroptotic	SLC1A5	[120]	↑ Ferroptosis
miR-7-5p	Anti-ferroptotic	ACSL4 (via ZFAS1)	[121]	↓ Ferroptosis
miR-873-5p	Anti-ferroptotic	HMOX1	[129]	↓ Ferroptosis
miR-223-3p	Anti-ferroptotic	ITPR3/GPX4/xCT	[123]	↓ Ferroptosis
miR-144-3p	Pro-ferroptotic	β-cell ferroptosis regulators	[128]	↑ Ferroptosis

#### MicroRNA and diabetic nephropathy (DN)

Furthermore, diabetic nephropathy (DN), a serious consequence of diabetes, has become more prevalent. Although Circular ASAP2 mediates DN, nothing is known about its biological mechanism and function. A study by Li and Meng [122] showed that circular ASAP2 protein declines the sex-determining region Y-Box 2 (SOX2), solute carrier family 7 member 11 (SLC7A11), and miR-770-5p activity, which increased in mice with DN. Thus, ASAP2 inhibition exacerbates DN and increases oxidative stress and inflammation.

A recent study explores the role of ferroptosis and the miR-223-3p/inositol 1,4,5-trisphosphate receptor type 3 (ITPR3) pathway in diabetic kidney disease (DKD) using adenoviruses. Overexpression or silencing of MiR-223-3p was accomplished with adenoviruses. The findings showed that elevated glucose levels lead to the downregulation of miR-223-3p. This downregulation is possibly linked to the regulation of ITPR3 with subsequent reduction of GPX4 and the cystine/glutamate transporter (xCT) and an increment in long-chain ACSL4, thereby enhancing ferroptosis. These changes result in increased calcium levels and alterations in iron metabolism. Thus, understanding the miR-3p/ITPR3 pathway in DKD, particularly in the context of adenoviral research, provides insights into potential therapeutic strategies. By targeting miR-223-3p/ITPR3/GPX4/xCT, the researchers successfully prevented high glucose-induced ferroptosis [123] (Table 2).

Furthermore, circular RNAs (circRNAs) are essential in many human diseases. However, their function in DKD is still unclear. Circ-0069561 was up-regulated in kidney tissues from DKD patients using high-throughput RNA sequencing. Fluorescent in situ hybridization (FISH) and real-time PCR were used to validate this RNA in DKD patients and type 2 diabetic mice. Moreover, the circ-0069561 expression was significantly elevated and primarily localized in the glomerulus in the mouse podocyte clone 5. A higher risk of primary endpoints was linked to high expression of circ-0069561. Circ-0069561 may be the perfect biomarker and treatment target for the advancement of DKD [124].

By analyzing m6A RNA methylation, another epigenetic mechanism, and its impact on ferroptosis-related gene expression in type 2 diabetes and its microvascular complications, the next part expands on the role of non-coding RNAs, such as circRNAs, in regulating ferroptosis in diabetic complications.

### Interplay between m6A RNA methylation and ferroptosis in diabetes development and therapy

N6-methyladenosine (m6A), a common RNA modulator, can affect critical genes related to ferroptosis, including cyclin-dependent kinase inhibitor 1A (CDKN1A), myo-inositol oxygenase (MIOX), proto-oncogene, BHLH transcription factor (MYCN), and cluster of differentiation 82 (CD82). They exhibit differential expression levels in diabetic conditions. These genes can negatively affect mitochondrial function, oxidative stress, and insulin secretion, all of which are crucial in the etiology of T2D. For example, MIOX overexpression during hyperglycemia accelerates ferroptosis and beta-cell damage by promoting lipid peroxidation and lowering antioxidant defense. Ferroptosis is further exacerbated by MYCN, which also alters redox balance and iron metabolism. The study reported that ferroptosis gene regulation by m6A may be a new target for diagnosis and treatment [125, 126].

Moreover, further research has demonstrated that the relationship between ferroptosis and m6A demethylation in the context of diabetic retinopathy (DR), a microvascular consequence of type 2 diabetes, using high-glucose-treated ARPE-19 cells and STZ-induced diabetic rats to examine the effect of alkylation repair homolog protein 5 (ALKBH5), a m6A demethylase, on ferroptosis in DR. The expression levels of ALKBH5, YTH N6-methyladenosine RNA binding protein 1 (YTHDF1), and ACSL4 were examined. The results showed that both models had elevated ferroptosis, with downregulated ALKBH5 and upregulated YTHDF1 and ACSL4. Interestingly, either YTHDF1 silencing or ALKBH5 expression restoration partially reversed ferroptosis. ALKBH5 mechanistically reduced the stability of ACSL4 mRNA through m6A demethylation in a manner that was reliant on YTHDF1. These results point to the m6A–YTHDF1–ACSL4 axis as a possible therapeutic target [127].

In addition, recent findings highlight miR-144-3p as a critical regulator of ferroptosis in pancreatic *β*-cells, linking non-coding RNA biology to diabetes pathogenesis. In T2DM model mice, which were induced by a high-fat diet combined with streptozotocin injection, miR-144-3p was up-regulated under hyperglycemic conditions, where it promoted *β*-cell ferroptosis and impaired insulin secretion by directly targeting ubiquitin-specific peptidase 22 (USP22) and destabilizing the USP22/SIRT1 axis. Inhibition of miR-144-3p, conversely, restored antioxidant defenses (GPX4, GSH), preserved β-cell viability, and improved glycemic control in vivo. These findings suggest that miR-144-3p acts as a pro-ferroptosis driver in islet dysfunction, and its targeting may represent a promising therapeutic avenue to preserve *β*-cell function in type 2 diabetes [128].

Recent studies have identified several miRNAs that modulate ferroptosis-related pathways in diabetic pathogenesis. Table 3 summarises the key miRNAs, their targets, and their functional roles in ferroptosis regulation.

**Table 3:** Regulatory miRNA of the ferroptosis pathway in type 2 diabetes (T2D)

Type of study model	Ferroptosis mechanism	Type of miRNA	Target action of miRNA	Evidence level	References
In vitro, RPE cells	-HG increased the levels of ROS after damaged mitochondria and GSSG, and improved the mitochondrial membrane’s lipid peroxidation density -Accumulation of iron and increased ferroptosis	miR-138-5p	-Increase the expression of Nrf2 and Sirt1, the antioxidant activity GPX4, GCLM, GCLC, and alleviate the DR	Preclinical	[116]
-In vivo study, C57BL/6 mice model	-HG increased the levels of ROS and lipid peroxide -Accumulation of iron and increased ferroptosis	miR-770-5p	-Decrease the SOX2 which can affect the SLC7A expression -Decrease the SLC7A and increase the oxidative stress damage	Preclinical	[122]
Bioinformatics study	-Increase the accumulated iron and lipid peroxides - p53 activation and regulation of ferroptosis	-Hsa-miR-873-5p	- Decrease the HMOX1 and PTGS2 expression	Computational detection	[118]
In vitro glomerular endothelial cells	-HG decreased the levels of miR-223-3p	-miR-223-3p	-Decrease the xCT and GPX4, and increase ACSL4 expression	Preclinical	[123]
In vitro retinal endothelial cells of diabetic human	-HG caused the ZFAS1 elevation	-miR-7-5p	- Regulating the expression of ACSL4	Preclinical	[121]
In vitro retinal epithelium cells	HG increased the levels of ROS and ferroptosis activation	-miR-338-3p	-Inhibition of SLC1A5	Preclinical	[120]
In vitro, ARPE19	HG increased the levels of ROS and ferroptosis activation	-miR-200b-3p	Target CFL2 gene	Preclinical	[117]

## Ferroptosis-modulating medications from the lab to the clinic

Preclinical studies show a strong link between ferroptosis and type 2 diabetes, with evidence mainly from in vitro experiments and animal models. Ferroptosis contributes to insulin resistance and pancreatic *β*-cell dysfunction, but extrapolating these findings to the clinic remains a challenge. Small-molecule ferroptosis inhibitors face difficulties in preclinical trials, including limited efficacy, poor pharmacokinetics, low stability, low solubility, low targeting, low safety, and toxicity [130].

### Challenges that preclinical experiments face

Without good diagnostic markers, it is difficult to monitor ferroptosis activity or assess therapeutic efficacy. In addition, most current investigations focus on single-target mechanisms, which may not capture the complexity of ferroptosis regulation in human metabolic disease. the clinical trials should be conducted to evaluate the safety and efficacy of ferroptosis inhibitors in T2D, develop markers of ferroptosis activity in diabetic tissues and investigate nanotechnology-based drug delivery techniques to enhance drug specificity and reduce toxicity. These challenges must be addressed if ferroptosis is to be used as a promising therapeutic target in the management of T2D.

Clinical evidence for treating T2D patients with ferroptosis-targeted therapy is still lacking. For instance, in animal models, Fer-1, the first synthetic inhibitor of ferroptosis, has been implicated in numerous diseases [131]. However, it is still in the experimental stage, which makes it challenging to transition to clinical trials.

In meantime, compound 51, a phenothiazine-based derivative, shows substantial hERG inhibitory activity despite having neuroprotective effects in IS animal models [132]. In a multicenter, randomised, double-blind, placebo-controlled trial of AD from 2007 to 2012, vitamin E was found to significantly reduce cognitive performance in patients with mild to moderate AD. On the other hand, some studies contend that vitamin E supplementation does not lower the risk of AD and slows down its pathogenesis [133]. Although iron chelators such as deferoxamine (DFO) and deferiprone (DFP) have been shown to reduce ferroptosis-mediated damage in other diseases, they have not been well-studied in diabetes in human subjects. Moreover, existing ferroptosis inhibitors are troubled by issues, such as short half-lives, poor bioavailability, and off-target effects, which compromise their therapeutic benefits. To overcome these restrictions, CN128, a novel oral iron chelator, has been created [134, 135].

While copper compound CuII (ATSM) has demonstrated neuroprotective effects in ALS and PD phase I studies, patients in the clinical trial phase have been discovered to have spinal cord immunocompromise. With Dabigatran in a Phase III clinical trial in IS and Argatroban in a Phase IV clinical trial, it has been discovered that thrombin inhibition reduces ferroptosis-mediated neurological damage. To treat ferroptosis-mediated diseases, decrease side effects, and increase treatment consumption, researchers must optimize medications [136].

## Conclusion

Disorders in iron metabolism led to inadequate insulin secretion and insulin resistance. However, the connection between iron metabolism and T2D, along with its consequences, remained ambiguous until the identification of ferroptosis. Elevated concentrations of free reactive iron induce tissue damage and oxidative cellular demise with subsequent ferroptosis. This process is controlled at several levels, including iron equilibrium, cells with mitochondria, glutathione–GPX4 axis, and polyunsaturated fatty acid metabolism. Ferroptosis susceptibility is modulated by miRNAs, one important component of pathophysiological disorders associated with iron overload is the dysregulation of miRNA-regulated ferroptosis. Astragaloside-IV, a naturally occurring substance from Astragalus downregulates miR-138-5p in RPE cells of the DR model, leading to an increase in the expression of Nrf2, Sirt1, GPX4, GCLM, and GCLC. MiRNAs as hsa-miR-873-5p, hsa-miR-624-5p, hsa-miR-542-3p, and miR-338-3p, are critical regulators of essential ferroptosis-related genes. ZFAS1, a long noncoding RNA, may regulate ACSL4 expression in ferroptosis in human retinal endothelial cells. Future studies should focus on developing miRNA-based diagnostic assays for early ferroptosis-associated T2D alterations, clinical trials targeting ferroptosis pathways discussing therapeutic interventions, exploring ferroptosis’s role in diabetic complications, and understanding the complex web of miRNA-dependent ferroptosis mechanisms for T2D diagnosis and therapy.

## Data Availability

No datasets were generated or analysed during the current study.

## Abbreviations

T2D: Type 2 diabetes
ROS: Reactive oxygen species
caspases: Aspartate-specific cysteine proteases
HbA1C: Glycated hemoglobin
IDF: International Diabetes Federation
m6A: N6-methyladenosine
IR: Insulin resistance
GLUT2: Glucose transporter 2
UPR: Unfolded protein response
ER: Endoplasmic reticulum
IAAP: Islet amyloid polypeptides
AGEs: Advanced glycation end products
NF-κB: Nuclear factor kappa B
RAS: Renin–angiotensin system
RCD: Regulated cell death
BAK: Bcl-2 homologous antagonist/killer
BAX: Bcl-2-associated X protein
GPX4: Glutathione peroxidase 4
TF: Transferrin
RIPK1: Receptor-interacting protein kinase 1
MLKL: Mixed lineage kinase domain-like protein
STEAP3: Six transmembrane epithelial antigens of prostate 3
DMT1: Divalent metal transporter 1
LIP: Labile Iron Pool
SLC7A11: Solute Carrier Family 7 Member 11
SLC3A2: Solute Carrier Family 3 Member 2
GCL: Glutamate–cysteine ligase
GSS: Glutathione
NADPH: Nicotinamide adenine dinucleotide phosphate
VDAC2/3: Voltage-dependent channel 2/3
PUFAs: Polyunsaturated fatty acids
LPCAT3: Lysophosphatidylcholine acyltransferase 3
PE: Acyl-CoA to membrane phosphatidylcholine
LOX: Lipoxygenase
LPOX: Lipid peroxidation
PHL-OOH: Phospholipid hydroperoxides
L-ROS: Lipid reactive oxygen species
PL-OH: Phospholipid alcohols
ALOX-12/15: Lipoxygenase 12/15
SOD: Superoxide dismutase
GSIS: Glucose-stimulated insulin secretion
AMPK: AMP-activated protein kinase
LKB1: Liver kinase B1
ACC1: Acetyl-CoA carboxylase 1
FAS: Fatty acid synthase
GABA: Gamma-aminobutyric acid
Nrf2: Nuclear factor erythroid 2-related factor 2
NAFLD: Non-alcoholic fatty liver disease
EPO: Erythropoietin, Pol: poliumoside
miRNAs: MicroRNAs
DR: Diabetic retinopathy
RPE: Retinal pigment epithelial
Sirt1: Sirtuin 1
GCLM: Glutamate–cysteine ligase modifier subunit
GCLC: Glutamate–cysteine ligase catalytic subunit
CFL2: Cofilin, TFR1: transferrin receptor 1
HMOX1: Heme oxygenase 1
PTGS2: Prostaglandin–endoperoxide synthase 2
ZFAS1: Zinc finger antisense 1
DN: Diabetic nephropathy
SOX2: Sex-determining region Y-Box 2
SLC7A11: Solute carrier family 7 Member 11
ITPR3: Inositol 1, 4, 5-trisphosphate receptor type 3
DKD: Diabetic kidney disease
circRNAs: Circular RNAs
m6A: N6-methyladenosine
CDKN1A: Cyclin-dependent kinase inhibitor 1A
MIOX: Myo-inositol oxygenase
MYCN: BHLH transcription factor
CD82: Cluster of differentiation 82
ALKBH5: Alkylation repair homolog protein 5

## References

[1] Karamanou M, Protogerou A, Tsoucalas G, Androutsos G, Poulakou-Rebelakou E. Milestones in the history of diabetes mellitus: the main contributors. World J Diabetes. 2016;7(1):1. 26788261 10.4239/wjd.v7.i1.1 PMC4707300

[2] Tönnies T, Brinks R, Isom S, Dabelea D, Divers J, Mayer-Davis EJ, et al. Projections of type 1 and type 2 diabetes burden in the US population aged< 20 years through 2060: the SEARCH for diabetes in youth study. Diabetes Care. 2023;46(2):313–20. 36580405 10.2337/dc22-0945 PMC9887625

[3] Control CfD Prevention. National diabetes statistics report 2020: estimates of diabetes and its burden in the United States. 2022; 2023.

[4] Zunic S, Zunic S. Feroptosis of alveolar macrophages in lung cancer. Eur Respiratory Soc. 2016. 10.1183/13993003.congress-2016.PA514.

[5] Chen X, Kang R, Kroemer G, Tang D. Ferroptosis in infection, inflammation, and immunity. J Exp Med. 2021;218(6):e20210518. 33978684 10.1084/jem.20210518 PMC8126980

[6] Ocansey DKW, Yuan J, Wei Z, Mao F, Zhang Z. Role of ferroptosis in the pathogenesis and as a therapeutic target of inflammatory bowel disease. Int J Mol Med. 2023;51(6):1–16. 37203397 10.3892/ijmm.2023.5256 PMC10198063

[7] Sha W, Hu F, Xi Y, Chu Y, Bu S. Mechanism of ferroptosis and its role in type 2 diabetes mellitus. J Diabetes Res. 2021;2021(1):9999612. 34258295 10.1155/2021/9999612 PMC8257355

[8] Hu Z, Zhang H, Yang S-k, Wu X, He D, Cao K, et al. Emerging role of ferroptosis in acute kidney injury. Oxid Med Cell Longev. 2019;2019(1):8010614. 31781351 10.1155/2019/8010614 PMC6875218

[9] Xie Y, Hou W, Song X, Yu Y, Huang J, Sun X, et al. Ferroptosis: process and function. Cell Death Differ. 2016;23(3):369–79. 26794443 10.1038/cdd.2015.158 PMC5072448

[10] Li J, Cao F, Yin H-l, Huang Z-j, Lin Z-t, Mao N, et al. Ferroptosis: past, present and future. Cell Death Dis. 2020;11(2):88. 32015325 10.1038/s41419-020-2298-2 PMC6997353

[11] Jin X, Tang J, Qiu X, Nie X, Ou S, Wu G, et al. Ferroptosis: emerging mechanisms, biological function, and therapeutic potential in cancer and inflammation. Cell Death Discov. 2024;10(1):45. 38267442 10.1038/s41420-024-01825-7 PMC10808233

[12] Gao H, Jin Z, Bandyopadhyay G, Wang G, Zhang D, Rocha KCE, et al. Aberrant iron distribution via hepatocyte-stellate cell axis drives liver lipogenesis and fibrosis. Cell Metab. 2022;34(8):1201–13. 35921818 10.1016/j.cmet.2022.07.006 PMC9365100

[13] Zhang Z, Funcke J-B, Zi Z, Zhao S, Straub LG, Zhu Y, et al. Adipocyte iron levels impinge on a fat-gut crosstalk to regulate intestinal lipid absorption and mediate protection from obesity. Cell Metab. 2021;33(8):1624–39. 34174197 10.1016/j.cmet.2021.06.001 PMC8338877

[14] Zaharia OP, Bobrov P, Strassburger K, Bodis K, Karusheva Y, Scholz M, et al. Metabolic characteristics of recently diagnosed adult-onset autoimmune diabetes mellitus. J Clin Endocrinol Metab. 2018;103(2):429–37. 29220505 10.1210/jc.2017-01706

[15] Drivsholm T, de Fine ON, Nielsen A, Siersma V. Symptoms, signs and complications in newly diagnosed type 2 diabetic patients, and their relationship to glycaemia, blood pressure and weight. Diabetologia. 2005;48:210–4. 15650820 10.1007/s00125-004-1625-y

[16] Marshall SM, Flyvbjerg A. Prevention and early detection of vascular complications of diabetes. BMJ. 2006;333(7566):475–80. 16946335 10.1136/bmj.38922.650521.80 PMC1557968

[17] Pasquel FJ, Umpierrez GE. Hyperosmolar hyperglycemic state: a historic review of the clinical presentation, diagnosis, and treatment. Diabetes Care. 2014;37(11):3124–31. 25342831 10.2337/dc14-0984 PMC4207202

[18] Fasanmade OA, Odeniyi IA, Ogbera AO. Diabetic ketoacidosis: diagnosis and management. Afr J Med Med Sci. 2008;37(2):99–105. 18939392

[19] DeFronzo RA, Ferrannini E, Groop L, Henry RR, Herman WH, Holst JJ, et al. Type 2 diabetes mellitus. Nat Rev Dis Primers. 2015;1(1):1–22. 10.1038/nrdp.2015.19 27189025

[20] Kulkarni P, Devkumar P, Chattopadhyay I. Could dysbiosis of inflammatory and anti-inflammatory gut bacteria have an implications in the development of type 2 diabetes? A pilot investigation. BMC Res Notes. 2021;14:1–7. 33549142 10.1186/s13104-021-05466-2 PMC7868023

[21] Pedersen HK, Gudmundsdottir V, Nielsen HB, Hyotylainen T, Nielsen T, Jensen BA, et al. Human gut microbes impact host serum metabolome and insulin sensitivity. Nature. 2016;535(7612):376–81. 27409811 10.1038/nature18646

[22] Mackin ST, Nelson SM, Kerssens JJ, Wood R, Wild S, Colhoun HM, et al. Diabetes and pregnancy: national trends over a 15 year period. Diabetologia. 2018;61(5):1081–8. 29322220 10.1007/s00125-017-4529-3 PMC6448996

[23] Tekumit H, Cenal AR, Polat A, Uzun K, Tataroglu C, Akinci E. Diagnostic value of hemoglobin A1c and fasting plasma glucose levels in coronary artery bypass grafting patients with undiagnosed diabetes mellitus. Ann Thorac Surg. 2010;89(5):1482–7. 20417764 10.1016/j.athoracsur.2009.11.033

[24] Asif M. The prevention and control the type-2 diabetes by changing lifestyle and dietary pattern. J Educ Health Promot. 2014;3(1):1. 24741641 10.4103/2277-9531.127541 PMC3977406

[25] Papanas N, Maltezos E. Metformin: a review of its use in the treatment of type 2 diabetes. Clinical Medicine Therapeutics. 2009. 10.4137/CMT.S1085.

[26] Gæde P, Vedel P, Larsen N, Jensen GV, Parving H-H, Pedersen O. Multifactorial intervention and cardiovascular disease in patients with type 2 diabetes. N Engl J Med. 2003;348(5):383–93. 12556541 10.1056/NEJMoa021778

[27] Zheng Y, Ley SH, Hu FB. Global aetiology and epidemiology of type 2 diabetes mellitus and its complications. Nat Rev Endocrinol. 2018;14(2):88–98. 29219149 10.1038/nrendo.2017.151

[28] Cerf ME. Beta cell dysfunction and insulin resistance. Front Endocrinol. 2013;4:37. 10.3389/fendo.2013.00037 PMC3608918 23542897

[29] Hu FB, Manson JE, Stampfer MJ, Colditz G, Liu S, Solomon CG, et al. Diet, lifestyle, and the risk of type 2 diabetes mellitus in women. N Engl J Med. 2001;345(11):790–7. 11556298 10.1056/NEJMoa010492

[30] Schellenberg ES, Dryden DM, Vandermeer B, Ha C, Korownyk C. Lifestyle interventions for patients with and at risk for type 2 diabetes: a systematic review and meta-analysis. Ann Intern Med. 2013;159(8):543–51. 24126648 10.7326/0003-4819-159-8-201310150-00007

[31] Stumvoll M, Goldstein BJ, Van Haeften TW. Type 2 diabetes: principles of pathogenesis and therapy. Lancet. 2005;365(9467):1333–46. 15823385 10.1016/S0140-6736(05)61032-X

[32] Weyer C, Bogardus C, Mott DM, Pratley RE. The natural history of insulin secretory dysfunction and insulin resistance in the pathogenesis of type 2 diabetes mellitus. J Clin Invest. 1999;104(6):787–94. 10491414 10.1172/JCI7231 PMC408438

[33] DeFronzo RA. From the triumvirate to the ominous octet: a new paradigm for the treatment of type 2 diabetes mellitus. Diabetes. 2009;58(4):773–95. 19336687 10.2337/db09-9028 PMC2661582

[34] Schwartz SS, Epstein S, Corkey BE, Grant SF, Gavin JR III, Aguilar RB. The time is right for a new classification system for diabetes: rationale and implications of the β-cell–centric classification schema. Diabetes Care. 2016;39(2):179–86. 26798148 10.2337/dc15-1585 PMC5317235

[35] Bunney P, Zink A, Holm A, Billington C, Kotz C. Orexin activation counteracts decreases in nonexercise activity thermogenesis (NEAT) caused by high-fat diet. Physiol Behav. 2017;176:139–48. 28363838 10.1016/j.physbeh.2017.03.040 PMC5510739

[36] Fu Z, Gilbert ER, Liu D. Regulation of insulin synthesis and secretion and pancreatic Beta-cell dysfunction in diabetes. Curr Diabetes Rev. 2013;9(1):25–53. 22974359 PMC3934755

[37] Boland BB, Rhodes CJ, Grimsby JS. The dynamic plasticity of insulin production in β-cells. Mol Metab. 2017;6(9):958–73. 28951821 10.1016/j.molmet.2017.04.010 PMC5605729

[38] Rorsman P, Ashcroft FM. Pancreatic β-cell electrical activity and insulin secretion: of mice and men. Physiol Rev. 2018;98(1):117–214. 29212789 10.1152/physrev.00008.2017 PMC5866358

[39] Galicia-Garcia U, Benito-Vicente A, Jebari S, Larrea-Sebal A, Siddiqi H, Uribe KB, et al. Pathophysiology of type 2 diabetes mellitus. Int J Mol Sci. 2020;21(17):6275. 32872570 10.3390/ijms21176275 PMC7503727

[40] Nishikawa T, Edelstein D, Du XL, Yamagishi S-i, Matsumura T, Kaneda Y, et al. Normalizing mitochondrial superoxide production blocks three pathways of hyperglycaemic damage. Nature. 2000;404(6779):787–90. 10783895 10.1038/35008121

[41] Kowluru RA, Abbas SN, Odenbach S. Reversal of hyperglycemia and diabetic nephropathy: effect of reinstitution of good metabolic control on oxidative stress in the kidney of diabetic rats. J Diabetes Complic. 2004;18(5):282–8. 10.1016/j.jdiacomp.2004.03.002 15337502

[42] Kowluru RA, Kanwar M, Kennedy A. Metabolic memory phenomenon and accumulation of peroxynitrite in retinal capillaries. J Diabetes Res. 2007. 10.1155/2007/21976. 10.1155/2007/21976 PMC1906703 17641740

[43] Ceriello A, Ihnat MA, Thorpe JE. The “metabolic memory”: is more than just tight glucose control necessary to prevent diabetic complications? J Clin Endocrinol Metab. 2009;94(2):410–5. 19066300 10.1210/jc.2008-1824

[44] Reddy MA, Natarajan R. Epigenetic mechanisms in diabetic vascular complications. Cardiovasc Res. 2011;90(3):421–9. 21266525 10.1093/cvr/cvr024 PMC3096305

[45] Miao R, Fang X, Zhang Y, Wei J, Zhang Y, Tian J. Iron metabolism and ferroptosis in type 2 diabetes mellitus and complications: mechanisms and therapeutic opportunities. Cell Death Dis. 2023;14(3):186. 36882414 10.1038/s41419-023-05708-0 PMC9992652

[46] Dolma S, Lessnick SL, Hahn WC, Stockwell BR. Identification of genotype-selective antitumor agents using synthetic lethal chemical screening in engineered human tumor cells. Cancer Cell. 2003;3(3):285–96. 12676586 10.1016/s1535-6108(03)00050-3

[47] Dixon SJ, Lemberg KM, Lamprecht MR, Skouta R, Zaitsev EM, Gleason CE, et al. Ferroptosis: an iron-dependent form of nonapoptotic cell death. Cell. 2012;149(5):1060–72. 22632970 10.1016/j.cell.2012.03.042 PMC3367386

[48] Fuchs Y, Steller H. Programmed cell death in animal development and disease. Cell. 2011;147(4):742–58. 22078876 10.1016/j.cell.2011.10.033 PMC4511103

[49] Thompson CB. Apoptosis in the pathogenesis and treatment of disease. Science. 1995;267(5203):1456–62. 7878464 10.1126/science.7878464

[50] Peña-Blanco A, García-Sáez AJ. Bax, Bak and beyond—mitochondrial performance in apoptosis. FEBS J. 2018;285(3):416–31. 28755482 10.1111/febs.14186

[51] Jiang L, Kon N, Li T, Wang SJ, Su T, Hibshoosh H, et al. Ferroptosis as a p53-mediated activity during tumour suppression. Nature. 2015;520(7545):57–62. 25799988 10.1038/nature14344 PMC4455927

[52] Yagoda N, von Rechenberg M, Zaganjor E, Bauer AJ, Yang WS, Fridman DJ, et al. RAS-RAF-MEK-dependent oxidative cell death involving voltage-dependent anion channels. Nature. 2007;447(7146):864–8. 17568748 10.1038/nature05859 PMC3047570

[53] Ni L, Yuan C, Wu X. Targeting ferroptosis in acute kidney injury. Cell Death Dis. 2022;13(2):182. 35210424 10.1038/s41419-022-04628-9 PMC8873203

[54] Chen L, Yin Z, Qin X, Zhu X, Chen X, Ding G, et al. CD74 ablation rescues type 2 diabetes mellitus-induced cardiac remodeling and contractile dysfunction through pyroptosis-evoked regulation of ferroptosis. Pharmacol Res. 2022;176:106086. 35033649 10.1016/j.phrs.2022.106086

[55] Tang W, Li Y, He S, Jiang T, Wang N, Du M, et al. Caveolin-1 alleviates diabetes-associated cognitive dysfunction through modulating neuronal ferroptosis-mediated mitochondrial homeostasis. Antioxid Redox Signal. 2022;37(13):867–86. 35350885 10.1089/ars.2021.0233

[56] Ye H, Wang R, Wei J, Wang Y, Zhang X, Wang L. Bioinformatics analysis identifies potential ferroptosis key gene in type 2 diabetic islet dysfunction. Front Endocrinol. 2022;13:904312. 10.3389/fendo.2022.904312 PMC9309693 35898457

[57] Hu Z-W, Chen L, Ma R-Q, Wei F-Q, Wen Y-H, Zeng X-L, et al. Comprehensive analysis of ferritin subunits expression and positive correlations with tumor-associated macrophages and T regulatory cells infiltration in most solid tumors. Aging. 2021;13(8):11491. 33864445 10.18632/aging.202841 PMC8109065

[58] Richardson DR, Ponka P. The molecular mechanisms of the metabolism and transport of iron in normal and neoplastic cells. Biochimica Et Biophysica Acta (BBA)-Rev Biomembr. 1997;1331(1):1–40. 10.1016/s0304-4157(96)00014-7 9325434

[59] Chen J, Wang Y, Wu J, Yang J, Li M, Chen Q. The potential value of targeting ferroptosis in early brain injury after acute CNS disease. Front Mol Neurosci. 2020;13:110. 32625062 10.3389/fnmol.2020.00110 PMC7314952

[60] Nemeth E, Tuttle MS, Powelson J, Vaughn MB, Donovan A, Ward DM, et al. Hepcidin regulates cellular iron efflux by binding to ferroportin and inducing its internalization. Science. 2004;306(5704):2090–3. 15514116 10.1126/science.1104742

[61] Kulaksiz H, Fein E, Redecker P, Stremmel W, Adler G, Cetin Y. Pancreatic b-cells express hepcidin, an iron-uptake regulatory peptide. J Endocrinol. 2008;197(2):241–50. 18434354 10.1677/JOE-07-0528

[62] Aigner E, Felder TK, Oberkofler H, Hahne P, Auer S, Soyal S, et al. Glucose acts as a regulator of serum iron by increasing serum hepcidin concentrations. J Nutr Biochem. 2013;24(1):112–7. 22819549 10.1016/j.jnutbio.2012.02.017

[63] Zeng F, Nijiati S, Tang L, Ye J, Zhou Z, Chen X. Ferroptosis detection: from approaches to applications. Angew Chem. 2023;135(35):e202300379. 10.1002/anie.202300379 36828775

[64] Zheng S, Guan X-Y. Ferroptosis: promising approach for cancer and cancer immunotherapy. Cancer Lett. 2023;561:216152. 37023938 10.1016/j.canlet.2023.216152

[65] Li Y, Yan J, Zhao Q, Zhang Y, Zhang Y. ATF3 promotes ferroptosis in sorafenib-induced cardiotoxicity by suppressing Slc7a11 expression. Front Pharmacol. 2022;13:904314. 36210815 10.3389/fphar.2022.904314 PMC9537618

[66] Ursini F, Maiorino M, Valente M, Ferri L, Gregolin C. Purification from pig liver of a protein which protects liposomes and biomembranes from peroxidative degradation and exhibits glutathione peroxidase activity on phosphatidylcholine hydroperoxides. Biochimica et Biophysica Acta (BBA). 1982;710(2):197–211. 7066358 10.1016/0005-2760(82)90150-3

[67] He J, Li Z, Xia P, Shi A, FuChen X, Zhang J, et al. Ferroptosis and ferritinophagy in diabetes complications. Mol Metab. 2022;60:101470. 35304332 10.1016/j.molmet.2022.101470 PMC8980341

[68] Vu NT, Kim M, Stephenson DJ, MacKnight HP, Chalfant CE. Ceramide kinase inhibition drives ferroptosis and sensitivity to cisplatin in mutant KRAS lung cancer by dysregulating VDAC-mediated mitochondria function. Mol Cancer Res. 2022;20(9):1429–42. 35560154 10.1158/1541-7786.MCR-22-0085 PMC9444881

[69] Dierge E, Debock E, Guilbaud C, Corbet C, Mignolet E, Mignard L, et al. Peroxidation of n-3 and n-6 polyunsaturated fatty acids in the acidic tumor environment leads to ferroptosis-mediated anticancer effects. Cell Metab. 2021;33(8):1701–15. 34118189 10.1016/j.cmet.2021.05.016

[70] Yuan H, Li X, Zhang X, Kang R, Tang D. Identification of ACSL4 as a biomarker and contributor of ferroptosis. Biochem Biophys Res Commun. 2016;478(3):1338–43. 27565726 10.1016/j.bbrc.2016.08.124

[71] Kagan VE, Mao G, Qu F, Angeli JPF, Doll S, Croix CS, et al. Oxidized arachidonic and adrenic PEs navigate cells to ferroptosis. Nat Chem Biol. 2017;13(1):81–90. 27842066 10.1038/nchembio.2238 PMC5506843

[72] Maiorino M, Conrad M, Ursini F. GPx4, lipid peroxidation, and cell death: discoveries, rediscoveries, and open issues. Antioxid Redox Signal. 2018;29(1):61–74. 28462584 10.1089/ars.2017.7115

[73] Ursini F, Maiorino M, Gregolin C. The selenoenzyme phospholipid hydroperoxide glutathione peroxidase. Biochimica et Biophysica Acta (BBA)-Gen Sub. 1985;839(1):62–70. 10.1016/0304-4165(85)90182-5 3978121

[74] Yang WS, SriRamaratnam R, Welsch ME, Shimada K, Skouta R, Viswanathan VS, et al. Regulation of ferroptotic cancer cell death by GPX4. Cell. 2014;156(1–2):317–31. 24439385 10.1016/j.cell.2013.12.010 PMC4076414

[75] Mead JF. Free radical mechanisms of lipid damage and consequences for cellular membranes. Cambridge: Academic Press; 1976.

[76] Kuhn H, Banthiya S, Van Leyen K. Mammalian lipoxygenases and their biological relevance. Biochimica et Biophysica Acta (BBA). 2015;1851(4):308–30. 25316652 10.1016/j.bbalip.2014.10.002 PMC4370320

[77] Ling FY, Stockwell BR. Transforming lipoxygenases: PE-specific enzymes in disguise. Cell. 2017;171(3):501–2. 29053966 10.1016/j.cell.2017.10.006 PMC5960801

[78] Maillard B, Ingold K, Scaiano J. Rate constants for the reactions of free radicals with oxygen in solution. J Am Chem Soc. 1983;105(15):5095–9.

[79] Yin H, Xu L, Porter NA. Free radical lipid peroxidation: mechanisms and analysis. Chem Rev. 2011;111(10):5944–72. 21861450 10.1021/cr200084z

[80] Cheng Z, Li Y. What is responsible for the initiating chemistry of iron-mediated lipid peroxidation: an update. Chem Rev. 2007;107(3):748–66. 17326688 10.1021/cr040077w

[81] Stockwell BR, Angeli JPF, Bayir H, Bush AI, Conrad M, Dixon SJ, et al. Ferroptosis: a regulated cell death nexus linking metabolism, redox biology, and disease. Cell. 2017;171(2):273–85. 28985560 10.1016/j.cell.2017.09.021 PMC5685180

[82] Hansen J, Moen I, Mandrup-Poulsen T. Iron: the hard player in diabetes pathophysiology. Acta Physiol. 2014;210(4):717–32. 10.1111/apha.12256 24521359

[83] Sun L, Zong G, Pan A, Ye X, Li H, Yu Z, et al. Elevated plasma ferritin is associated with increased incidence of type 2 diabetes in middle-aged and elderly Chinese adults. J Nutr. 2013;143(9):1459–65. 23902953 10.3945/jn.113.177808

[84] Huang J, Jones D, Luo B, Sanderson M, Soto J, Abel ED, et al. Iron overload and diabetes risk: a shift from glucose to fatty acid oxidation and increased hepatic glucose production in a mouse model of hereditary hemochromatosis. Diabetes. 2011;60(1):80–7. 20876715 10.2337/db10-0593 PMC3012200

[85] Wang J, Wang H. Oxidative stress in pancreatic beta cell regeneration. Oxid Med Cell Longev. 2017;2017(1):1930261. 28845211 10.1155/2017/1930261 PMC5560096

[86] Bruni A, Pepper AR, Pawlick RL, Gala-Lopez B, Gamble AF, Kin T, et al. Ferroptosis-inducing agents compromise in vitro human islet viability and function. Cell Death Dis. 2018;9(6):595. 29789532 10.1038/s41419-018-0506-0 PMC5964226

[87] Luo E-F, Li H-X, Qin Y-H, Qiao Y, Yan G-L, Yao Y-Y, et al. Role of ferroptosis in the process of diabetes-induced endothelial dysfunction. World J Diabetes. 2021;12(2):124. 33594332 10.4239/wjd.v12.i2.124 PMC7839168

[88] Wei S, Qiu T, Yao X, Wang N, Jiang L, Jia X, et al. Arsenic induces pancreatic dysfunction and ferroptosis via mitochondrial ROS-autophagy-lysosomal pathway. J Hazard Mater. 2020;384:121390. 31735470 10.1016/j.jhazmat.2019.121390

[89] Du J, Wang T, Li Y, Zhou Y, Wang X, Yu X, et al. DHA inhibits proliferation and induces ferroptosis of leukemia cells through autophagy dependent degradation of ferritin. Free Radic Biol Med. 2019;131:356–69. 30557609 10.1016/j.freeradbiomed.2018.12.011

[90] Zhang Z, Li L, Fu W, Fu Z, Si M, Wu S, et al. Therapeutic effects of natural compounds against diabetic complications via targeted modulation of ferroptosis. Front Pharmacol. 2024;15:1425955. 39359249 10.3389/fphar.2024.1425955 PMC11445066

[91] Yi H, Peng H, Wu X, Xu X, Kuang T, Zhang J, et al. The therapeutic effects and mechanisms of quercetin on metabolic diseases: pharmacological data and clinical evidence. Oxid Med Cell Longev. 2021;2021(1):6678662. 34257817 10.1155/2021/6678662 PMC8249127

[92] Foretz M, Guigas B, Viollet B. Metformin: update on mechanisms of action and repurposing potential. Nat Rev Endocrinol. 2023;19(8):460–76. 37130947 10.1038/s41574-023-00833-4 PMC10153049

[93] Hsu S-K, Cheng K-C, Mgbeahuruike MO, Lin Y-H, Wu C-Y, Wang H-MD, et al. New insight into the effects of metformin on diabetic retinopathy, aging and cancer: nonapoptotic cell death, immunosuppression, and effects beyond the AMPK pathway. Int J Mol Sci. 2021;22(17):9453. 34502359 10.3390/ijms22179453 PMC8430477

[94] Lee H, Zandkarimi F, Zhang Y, Meena JK, Kim J, Zhuang L, et al. Energy-stress-mediated AMPK activation inhibits ferroptosis. Nat Cell Biol. 2020;22(2):225–34. 32029897 10.1038/s41556-020-0461-8 PMC7008777

[95] Yue F, Shi Y, Wu S, Xing L, He D, Wei L, et al. Metformin alleviates hepatic iron overload and ferroptosis through AMPK-ferroportin pathway in HFD-induced NAFLD. IScience. 2023. 10.1016/j.isci.2023.108560. 38089577 10.1016/j.isci.2023.108560 PMC10711470

[96] Ma W-Q, Sun X-J, Zhu Y, Liu N-F. Metformin attenuates hyperlipidaemia-associated vascular calcification through anti-ferroptotic effects. Free Radic Biol Med. 2021;165:229–42. 33513420 10.1016/j.freeradbiomed.2021.01.033

[97] Wang F, Liu X, Huang F, Zhou Y, Wang X, Song Z, et al. Gut microbiota-derived gamma-aminobutyric acid from metformin treatment reduces hepatic ischemia/reperfusion injury through inhibiting ferroptosis. Elife. 2024. 10.7554/eLife.89045.4. 38488837 10.7554/eLife.89045 PMC10942780

[98] Stielow M, Fijałkowski Ł, Alaburda A, Grześk G, Grześk E, Nowaczyk J, et al. SGLT2 inhibitors: from molecular mechanisms to clinical outcomes in cardiology and diabetology. Molecules. 2025;30(15):3112. 40807290 10.3390/molecules30153112 PMC12348473

[99] Han J-x, Luo L-l, Wang Y-c, Miyagishi M, Kasim V, Wu S-r. SGLT2 inhibitor empagliflozin promotes revascularization in diabetic mouse hindlimb ischemia by inhibiting ferroptosis. Acta Pharmacol Sin. 2023;44(6):1161–74. 36509902 10.1038/s41401-022-01031-0 PMC10203292

[100] Ma S, He L-L, Zhang G-R, Zuo Q-J, Wang Z-L, Zhai J-L, et al. Canagliflozin mitigates ferroptosis and ameliorates heart failure in rats with preserved ejection fraction. Naunyn-Schmiedebergs Arch Pharmacol. 2022;395(8):945–62. 35476142 10.1007/s00210-022-02243-1 PMC9276585

[101] Packer M. Potential interactions when prescribing SGLT2 inhibitors and intravenous iron in combination in heart failure. Heart Fail. 2023;11(1):106–14. 10.1016/j.jchf.2022.10.004 36396554

[102] Pandey A, Rath G, Chawala R, Goyal AK. A comprehensive review on liraglutide and novel nanocarrier-based systems for the effective delivery of liraglutide. Naunyn-Schmiedebergs Arch Pharmacol. 2025. 10.1007/s00210-025-03918-1. 40014122 10.1007/s00210-025-03918-1

[103] Song J-X, An J-R, Chen Q, Yang X-Y, Jia C-L, Xu S, et al. Liraglutide attenuates hepatic iron levels and ferroptosis in db/db mice. Bioengineered. 2022;13(4):8334–48. 35311455 10.1080/21655979.2022.2051858 PMC9161873

[104] An J-R, Su J-N, Sun G-Y, Wang Q-F, Fan Y-D, Jiang N, et al. Liraglutide alleviates cognitive deficit in db/db mice: involvement in oxidative stress, iron overload, and ferroptosis. Neurochem Res. 2022;47(2):279–94. 34480710 10.1007/s11064-021-03442-7

[105] Guo T, Yan W, Cui X, Liu N, Wei X, Sun Y, et al. Liraglutide attenuates type 2 diabetes mellitus-associated non-alcoholic fatty liver disease by activating AMPK/ACC signaling and inhibiting ferroptosis. Mol Med. 2023;29(1):132. 37770820 10.1186/s10020-023-00721-7 PMC10540362

[106] Li D, Jiang C, Mei G, Zhao Y, Chen L, Liu J, et al. Quercetin alleviates ferroptosis of pancreatic β cells in type 2 diabetes. Nutrients. 2020;12(10):2954. 32992479 10.3390/nu12102954 PMC7600916

[107] Yan W, Guo T, Liu N, Cui X, Wei X, Sun Y, et al. Erythropoietin ameliorates cognitive deficits by improving hippocampal and synaptic damage in streptozotocin-induced diabetic mice. Cell Signal. 2023;106:110614. 36739954 10.1016/j.cellsig.2023.110614

[108] Guo T, Yu Y, Yan W, Zhang M, Yi X, Liu N, et al. Erythropoietin ameliorates cognitive dysfunction in mice with type 2 diabetes mellitus via inhibiting iron overload and ferroptosis. Exp Neurol. 2023;365:114414. 37075971 10.1016/j.expneurol.2023.114414

[109] Deng R, Xu Y, Feng F, Liu W. Identification of poliumoside metabolites in rat feces by high performance liquid chromatography coupled with quadrupole time-of-flight tandem mass spectrometry. J Chromatogr B. 2014;969:285–96. 10.1016/j.jchromb.2014.08.032 25215644

[110] Zheng J-N, Zhuo J-Y, Nie J, Liu Y-L, Chen B-Y, Wu A-Z, et al. Phenylethanoid glycosides from callicarpa kwangtungensis chun attenuate TNF-α-Induced cell damage by inhibiting NF-κB pathway and enhancing Nrf2 pathway in A549 cells. Front Pharmacol. 2021;12:693983. 34305604 10.3389/fphar.2021.693983 PMC8293607

[111] Xu C-Y, Xu C, Xu Y-N, Du S-Q, Dai Z-H, Jin S-Q, et al. Poliumoside protects against type 2 diabetes-related osteoporosis by suppressing ferroptosis via activation of the Nrf2/GPX4 pathway. Phytomedicine. 2024;125:155342. 38295665 10.1016/j.phymed.2024.155342

[112] Lippi G, Franchini M, Favaloro EJ. Thrombotic complications of erythropoiesis-stimulating agents. Semin Thromb Hemost. 2010;36(05):537–49. 20632251 10.1055/s-0030-1255448

[113] Li M, Marin-Muller C, Bharadwaj U, Chow KH, Yao Q, Chen C. MicroRNAs: control and loss of control in human physiology and disease. World J Surg. 2009;33(4):667–84. 19030926 10.1007/s00268-008-9836-x PMC2933043

[114] Jin S, Liu P-S, Zheng D, Xie X. The interplay of miRNAs and ferroptosis in diseases related to iron overload. Apoptosis. 2024;29(1):45–65. 37758940 10.1007/s10495-023-01890-w

[115] Yaribeygi H, Katsiki N, Behnam B, Iranpanah H, Sahebkar A. Micrornas and type 2 diabetes mellitus: molecular mechanisms and the effect of antidiabetic drug treatment. Metabolism. 2018;87:48–55. 30253864 10.1016/j.metabol.2018.07.001

[116] Tang X, Li X, Zhang D, Han W. Astragaloside-IV alleviates high glucose-induced ferroptosis in retinal pigment epithelial cells by disrupting the expression of miR-138-5p/Sirt1/Nrf2. Bioengineered. 2022;13(4):8238–53. 10.1080/21655979.2022.2049471 PMC9162003 35302431

[117] Zhu Z, Duan P, Song H, Zhou R, Chen T. Downregulation of circular RNA PSEN1 ameliorates ferroptosis of the high glucose treated retinal pigment epithelial cells via miR-200b-3p/cofilin-2 axis. Bioengineered. 2021;12(2):12555–67. 34903141 10.1080/21655979.2021.2010369 PMC8809929

[118] Huang Y, Peng J, Liang Q. Identification of key ferroptosis genes in diabetic retinopathy based on bioinformatics analysis. PLoS ONE. 2023;18(1):e0280548. 36689408 10.1371/journal.pone.0280548 PMC9870164

[119] Alrashed MM, Alharbi H, Alshehry AS, Ahmad M, Aloahd MS. MiR-624-5p enhances NLRP3 augmented gemcitabine resistance via EMT/IL-1β/Wnt/β-catenin signaling pathway in ovarian cancer. J Reprod Immunol. 2022;150:103488. 35124344 10.1016/j.jri.2022.103488

[120] Zhou J, Sun C, Dong X, Wang H. A novel miR-338-3p/SLC1A5 axis reprograms retinal pigment epithelium to increases its resistance to high glucose-induced cell ferroptosis. J Mol Histol. 2022;53(3):561–71. 35320491 10.1007/s10735-022-10070-0

[121] Liu Y, Zhang Z, Yang J, Wang J, Wu Y, Zhu R, et al. lncRNA ZFAS1 Positively Facilitates Endothelial Ferroptosis via miR-7-5p/ACSL4 Axis in Diabetic Retinopathy. Oxid Med Cell Longev. 2022;2022(1):9004738. 36092160 10.1155/2022/9004738 PMC9453005

[122] Li Q, Meng X, Hua Q. Circ ASAP2 decreased inflammation and ferroptosis in diabetic nephropathy through SOX2/SLC7A11 by miR-770-5p. Acta Diabetol. 2023;60(1):29–42. 36153434 10.1007/s00592-022-01961-5

[123] Wang D, Zhang L, Nan J, Wan S, Luo J, Li X, et al. High glucose elevates intracellular calcium level and induces ferroptosis in glomerular endothelial cells through the miR-223-3p/ITPR3 pathway. Mol Cell Endocrinol. 2024;594:112384. 39426490 10.1016/j.mce.2024.112384

[124] Chen C, Liu X, Zhu S, Wang Y, Ma Y, Hu Z, et al. Circ-0069561 as a novel diagnostic biomarker for progression of diabetic kidney disease. Ren Fail. 2025;47(1):2490200. 40260530 10.1080/0886022X.2025.2490200 PMC12016256

[125] Cheng Y, Wan J, Xu Y, Liu S, Li L, Zhou J, et al. RBM15 promotes hypoxia/reoxygenation-induced ferroptosis in human cardiomyocytes by mediating m6A modification of ACSL4. Hereditas. 2025;162(1):135. 40682199 10.1186/s41065-025-00453-0 PMC12273425

[126] Wang J, Li X, Geng J, Wang R, Ma G, Liu P. Identification of biomarkers and mechanism exploration of ferroptosis related genes regulated by m6A in type 2 diabetes mellitus. Hereditas. 2025;162(1):24. 39966875 10.1186/s41065-025-00385-9 PMC11834627

[127] Liao Q, Li Y, Cui M, Liu M. m6A demethylase ALKBH5 reduces ferroptosis in diabetic retinopathy through the m6A-YTHDF1-ACSL4 axis. Diabet Med. 2025. 10.1111/dme.70033. 40210448 10.1111/dme.70033

[128] Zhang S, Liu X, Wang J, Yuan F, Liu Y. Targeting ferroptosis with miR-144-3p to attenuate pancreatic β cells dysfunction via regulating USP22/SIRT1 in type 2 diabetes. Diabetol Metab Syndr. 2022;14(1):89. 35761309 10.1186/s13098-022-00852-7 PMC9235078

[129] Zhao C, Wang T, Lu Y, Zhou Y, Chen J, Ju R. Copper-overload promotes ferroptosis in cervical cancer cells by upregulating HMOX1 expression. Discover Oncology. 2025;16(1):1549. 40810778 10.1007/s12672-025-03421-2 PMC12354449

[130] Guo X, Jin X, Han K, Kang S, Tian S, Lv X, et al. Iron promotes neurological function recovery in mice with ischemic stroke through endogenous repair mechanisms. Free Radic Biol Med. 2022;182:59–72. 35202785 10.1016/j.freeradbiomed.2022.02.017

[131] Wang Y, Lv M-n, Zhao W-j. Research on ferroptosis as a therapeutic target for the treatment of neurodegenerative diseases. Ageing Res Rev. 2023;91:102035. 37619619 10.1016/j.arr.2023.102035

[132] You J, Yang W, Ma R, Xia A, Zhang G, Fang Z, et al. Discovery of 2-vinyl-10H-phenothiazine derivatives as a class of ferroptosis inhibitors with minimal human Ether-a-go-go related gene (hERG) activity for the treatment of DOX-induced cardiomyopathy. Bioorg Med Chem Lett. 2022;74:128911. 35907606 10.1016/j.bmcl.2022.128911

[133] Ashraf A, So P-W. Spotlight on ferroptosis: iron-dependent cell death in Alzheimer’s disease. Front Aging Neurosci. 2020;12:196. 32760266 10.3389/fnagi.2020.00196 PMC7371849

[134] Lecornec N, Castex MP, Réguerre Y, Moreau P, Marie I, Garçon L, et al. Agranulocytosis in patients with Diamond-Blackfan anaemia (DBA) treated with deferiprone for post-transfusion iron overload: a retrospective study of the French DBA cohort. Br J Haematol. 2022. 10.1111/bjh.18366. 35852515 10.1111/bjh.18366

[135] Chen W, Yuan X, Li Z, Lu Z, Kong S, Jiang H, et al. CN128: a new orally active hydroxypyridinone iron chelator. J Med Chem. 2020;63(8):4215–26. 32208614 10.1021/acs.jmedchem.0c00137

[136] Zhang L, Luo YL, Xiang Y, Bai XY, Qiang RR, Zhang X, et al. Ferroptosis inhibitors: past, present and future. Front Pharmacol. 2024;15:1407335. 38846099 10.3389/fphar.2024.1407335 PMC11153831

